# Metal ions and graphene-based compounds as alternative treatment options for burn wounds infected by antibiotic-resistant *Pseudomonas aeruginosa*

**DOI:** 10.1007/s00203-019-01803-z

**Published:** 2020-01-08

**Authors:** Nathalie Karaky, Andrew Kirby, Andrew J. McBain, Jonathan A. Butler, Mohamed El Mohtadi, Craig E. Banks, Kathryn A. Whitehead

**Affiliations:** 1 Microbiology at Interfaces, Manchester Metropolitan University, Chester Street, Manchester, M1 5GD UK; 2 Leeds Institute of Medical Research, University of Leeds, Leeds, LS2 9JT UK; 3 Division of Pharmacy and Optometry, Faculty of Biology, Medicine and Health, The University of Manchester, Oxford Road, Manchester, M13 9PT UK; 4 Faculty of Science and Engineering, Manchester Metropolitan University, Chester Street, Manchester, M1 5GD UK

**Keywords:** Graphene oxide, Metal ions, Antibiotic resistance, Burns, *P. aeruginosa*

## Abstract

Burn infections caused by *Pseudomonas aeruginosa* pose a major complication in wound healing. This study aimed to determine the antimicrobial effect of metal ions, graphene (Gr), and graphene oxide (GO), individually and in combination, against the planktonic and biofilm states of two antimicrobially resistant clinical strains of *P. aeruginosa* each with different antibiotic resistance profiles. Minimum inhibitory, minimum bactericidal, and fractional inhibitory concentrations were performed to determine the efficacy of the metal ions and graphene composites individually and their synergy in combination. Crystal violet biofilm and XTT assays measured the biofilm inhibition and metabolic activity, respectively. Molybdenum, platinum, tin, gold, and palladium ions exhibited the greatest antimicrobial activity (MIC = 7.8–26.0 mg/L), whilst GO and Gr demonstrated moderate-to-no effect against the planktonic bacterial cells, irrespective of their antibiograms. Biofilms were inhibited by zinc, palladium, silver, and graphene. In combination, silver–graphene and molybdenum–graphene inhibited both the planktonic and biofilm forms of the bacteria making them potential candidates for development into topical antimicrobials for burns patients infected with antibiotic-resistant *P. aeruginosa*.

## Introduction

The burden of antimicrobial resistance is a daily challenge in clinical settings, especially in acute and intensive care units. *Pseudomonas aeruginosa* is one of the most clinically important antibiotic-resistant Gram-negative bacteria (AMR-GNB), being a common cause of urinary tract and lower respiratory tract infections, i.e., ventilator-associated pneumonia (Ruppé et al. 2015). It is also commonly found in burn infections (Coetzee et al. 2013). *P. aeruginosa* maintains chronic infection through the formation of biofilms, which causes a delay in burn wound healing, patient recovery, and it is also a causative agent of graft loss. (Coetzee et al. 2013; Gonzalez et al. 2016). Treating MDR *Pseudomonas* species infections is challenging, and alternatives to systemic administered antibiotics are needed. Silver impregnated dressings and topical chemotherapeutics, such as mafenide acetate 11.2% cream, are being clinically used in the treatment of superficial and deep burn wounds, with silver sulfadiazine (SSD) remaining the standard topical antimicrobial for burn infections (Adhya et al. 2015). However, following the excessive and uncontrolled use of silver in medical and non-medical applications, serious concerns have been raised regarding bacterial resistance to silver (Chopra 2007), and thus, there is a need to look for other silver combinations and metal mixtures that have the potential to be used as antimicrobials.

For the last decade, carbon nanostructures (CNS) have gained a significant attention due to their physical and chemical properties, being used in biosensors and coating biomaterials for tissue transplantation (Al-Jumaili et al. 2017). Several studies have shown that graphene-based materials exhibit antimicrobial activities (Dizaj et al. 2015; Ji et al. 2016; Slate et al. 2018). Graphene oxide and reduced graphene oxide have been previously demonstrated to inhibit the growth of *Escherichia coli* and *P. aeruginosa* (Hu et al. 2010; Gurunathan et al. 2012; Tu et al. 2013; Hussein-Al-Ali et al. 2014). However, the activity of graphene against *P. aeruginosa* when combined with metal ions is still not well documented.

In light of the increasing evidence of the antimicrobial resistance within bacterial isolates, including *P. aeruginosa*, there is a need for new antimicrobial and biocidal agents to be developed. We have, therefore, assessed the antimicrobial activity of 18 metal ions, graphene, and graphene oxide, individually, and in synergy against two clinically relevant AMR strains of *P. aeruginosa* with distinct antibiotic resistance profiles.

## Materials and methods

### Bacterial strains

This study evaluated two new clinical strains of *P. aeruginosa* (NK-1 and NK-2), which were cultured on tryptone soy agar (TSA) or broth (TSB) (Oxoid, UK) and incubated for 24 h at 37 °C in aerobic conditions. The isolates were collected from clinical samples from The Royal Bolton Hospital (Bolton, UK) from patient wounds. These bacteria were used as representative microorganisms recovered from wounds. *Staphylococcus epidermidis* ATCC 12228 and *S. epidermidis* ATCC 35984 served as biofilm negative and positive controls, respectively, and were cultured as above.

### Antibiotic susceptibility testing

Following the recommendations of the European Committee on Antimicrobial Susceptibility Testing (EUCAST) (EUCAST 2019), both *P. aeruginosa* isolates were tested against the following antibiotic discs using the disc diffusion method. The multidrug discs contained ciprofloxacin (5 µg), piperacillin (100 µg), imipenem (10 µg), cefepime (30 µg), fosfomycin (200 µg), colistin (10 µg), and tobramycin (10 µg) (AB Biodisk, UK). The zones of inhibition (ZoI) were measured in millimetres following 12–18 h incubation at 37 °C, and interpreted in accordance with the manufacturer’s recommendations. Bacterial isolates were designated as antibiotic resistant (AMR) if they were resistant to multiple antimicrobial agents, classes, or subclasses of antibiotics as defined by EUCAST (Magiorakos et al. 2012).

### Antimicrobial compounds

The metal ions evaluated in this study were Atomic Absorption Standards (AAS—1000 mg/L) (Sigma-Aldrich, UK). The metal ions, along with acid controls, were molybdenum (Mo) and tin (Sn) (in 10% HCl), rhodium (Rh), platinum (Pt), gold (Au), palladium (Pd), ruthenium (Ru) (in 5% HCl), gallium (Ga) (in 5% HNO_3_), zirconium (Zr), yttrium (Y), rhenium (Re), indium (I), aluminium (Al), zinc (Zn), tantalum (Ta), niobium (Nb), copper (Cu), and silver (Ag) (in 2% HNO_3_). Graphene compounds were mechanically ground into particles (200 nm–1 µm) and suspended in water. Graphene oxide of flake sizes (300–700 nm) in solution (500 mg/L) (Graphene-Supermarket, USA) was also evaluated). Test compounds were tested individually and in combination with either graphene (Gr) or graphene oxide (GO). The graphene oxide was synthesised and characterised according to the previously described method by Whitehead et al. (2017).

### Minimum inhibitory concentration (MIC) and minimum bactericidal concentration (MBC) assays

Bacterial species were grown overnight in Tryptone Soya Broth. Following a 10 min centrifugation at 1721×*g*, the supernatant was discarded, and the pellet re-suspended in 10 mL double strength TSB. The cultures were adjusted to an optical density (OD 600) of 1.0 (± 0.1) equivalent to approximately 1.0 × 10^8^ colony forming units/mL (CFU/mL) at 540 nm. In a 96-well microplate, 100 µL of TSB were added to all the wells. This was followed by the addition of 100 µL of the test compound to the first column. The volume was mixed thoroughly by aspiration, and 100 µL was removed and added to the second column. This step was repeated until the tenth column where 100 µL was discarded. Finally, 100 µL of bacterial culture mixed with 0.15% triphenyl blue chloride (TBC) and added to the first ten columns. The last two columns, serving as controls, were inoculated, respectively, with 100 µL of double strength TSB broth containing 0.15% TBC and 100 µL of TSB (0.15% TBC) with adjusted bacterial suspension. Microplates were sealed with Parafilm^®^ and incubated overnight at 37 °C. The first well displaying no blue pigmentation was designated as the MIC. To determine the MBC, 20 µL of the wells showing no blue colour were plated on TSA and incubated at 37 °C for 24 h. The lowest concentration showing no bacterial growth was defined as the MBC. The antimicrobial activity was considered to be substantial when MIC or MBC ≤ 30 mg/L, good antimicrobial activity if 31 mg/L ≤ MIC or MBC ≤ 60 mg/L, a moderate antimicrobial activity if 61 ≤ MIC or MBC ≤ 90, while low or no antimicrobial activity was assigned for MIC or MBC ≥ 91 mg/L.

### Fractional inhibitory concentration assay

The fractional inhibitory concentration (FIC) was used to establish the synergistic relationship between metal ions and graphene or graphene oxide in a 1:1 ratio, following the same steps described earlier in Sect. 2.4. FIC ratios of two compounds X and Y were calculated and interpreted as follows:

$$\sum {\text{FIC}}\, = \,{\text{FIC }}\left( X \right)\, + \,{\text{FIC }}\left( Y \right),$$

$$\begin{aligned} &{\text{FIC }}\;{\text{of}} \;{\text{compound}} \;X \\&\quad = \frac{{{\text{MIC}}\; {\text{of}}\; {\text{compound }}\;X \;{\text{in}}\; {\text{combination}}\; {\text{with}}\; Y}}{{{\text{MIC}}\; {\text{of}}\; {\text{compound}}\; X\; {\text{alone}}}}, \end{aligned}$$

$${\text{FIC}}\; {\text{of}} \, {\text{compound}}\; Y = \frac{{{\text{MIC}}\; {\text{of}} \, {\text{compound}} \, Y\; {\text{in}}\; {\text{combination }}\;{\text{with}} \, X}}{{{\text{MIC}}\; {\text{of}}\; {\text{compound}}\; Y\; {\text{alone}}}}.$$

The two tested compounds were designated as synergistic if ∑FIC ≤ 0.5; additive if 0.5 < ∑FIC ≤ 1; indifferent if ∑FIC > 1 and antagonistic if ∑FIC ≥ 4 (Jenkins and Schuetz 2012).

### Crystal violet biofilm assay

Fine polished 304 grade stainless steel coupons (10 mm × 10 mm) were used to assess biofilm formation in this study (Vaidya et al. 2017). The coupons were cleaned for 10 min in undiluted acetone, methanol, and ethanol (BDH, UK), respectively, and were washed with sterile water between each step. After drying, the coupons were placed into the centres of the wells of 12-well culture plates containing 1 mL of an adjusted bacterial cell suspension (OD 1.0). The plate was sealed with parafilm and incubated for 1 week at 37 °C under static conditions. After incubation, the coupons were washed gently with 2 mL of sterile distilled water to eradicate any loose planktonic cells, and then air-dried at room temperature for 2 h. One millilitre of each tested compound (500 mg/L) was added into the respective well and incubated overnight at 37 °C. For synergy testing of metal ions and graphene/graphene oxide, 500 µL of each compound were added. The tested compounds were disposed and the coupons were carefully washed with 1 mL of sterile distilled water to remove non-adherent cells. Adherent cells were stained with 0.03% crystal violet (Oxoid, UK) for 30 min, and then washed with sterile water and left to air-dry for 1 h. One millilitre of 33% glacial acetic acid (BDH, UK) was added to each well. After 30 min, the supernatant was removed and the absorbance was read at OD_590_. Wells incubated with TSB only served as controls and were used to assess the adhesion ability of the biofilm. The biofilm forming strength was evaluated by comparing the OD_sample_ to the OD_blank_ (containing sterile TSB only). The subsequent classification was applied, adapted from Xu et al. (2016): weak biofilm formation (OD_b_ < OD_s_ ≤ 2 OD_b_), moderate (OD_b_ < OD_s_ ≤ 4 OD_b_), and strong biofilm formation (4 OD_b_ ≤ OD_s_).

### XTT assay

Before setting the experiment, the XTT solution was prepared by dissolving 4 mg of XTT (2,3-bis (2-methoxy-4-nitro-5-sulfophenyl)-5-[(phenylamino) carbonyl] 2H-tetrazolium hydroxide) (Sigma, UK) in 10 mL of phosphate buffer saline pre-warmed to 37 °C. Subsequently, 1.5 mL of the previously prepared XTT added to 300 µL of menadione solution (0.4 mM in acetone) to prepare the XTT-menadione solution. The bacterial biofilms were formed for 7 days on the stainless steel coupons as previously described in CVBA assay. On the 7th day, the bacterial supernatant was removed and coupons were washed thrice with distilled water. The tested compounds were added to the designated wells at 2 × MIC and were incubated for 1 h at 37 °C in static conditions. After washing the biofilms three times with PBS, the XTT-menadione solution (750 µL) were added to each well and incubated in the dark at 37 °C. After 3 h, the OD of the supernatant was read at 490 nm using a spectrophotometer. Blanks constituted of sterile medium with XTT/menadione solution and their values were directly deducted from the sample values to minimize background intervention. Cells untreated with the tested compounds and cells treated with 70% isopropyl alcohol were set as negative and positive controls, respectively. The change in the metabolic activity in the biofilm cells was calculated as percentage of reduction using the following formula:

$$\left[ {\frac{{{\text{OD growth control}} - {\text{OD sample}}}}{\text{OD growth conrol}}} \right] \times 100.$$

### Statistical analysis

All assays were carried out in triplicate per strain (*n* = 3). Independent sample *t* tests with a two-tailed distribution and the one-way analysis of variance test (ANOVA) with post hoc test were performed to assess the significance of the different treatments using GraphPad Prism 7.00 Software, Inc. and IBM SPSS Statistics (version 25). The distribution of the data from the mean values was analysed using standard deviation and error with 95% confidence intervals (Microsoft Excel). *p *< 0.05 was considered significant.

## Results

### Antimicrobial susceptibility testing

The two bacterial isolates displayed different antibiotic susceptibility patterns, and both were shown to be antibiotic resistant (AMR). *P. aeruginosa*-NK1 was found to be susceptible to cefepime, colistin, fosfomycin, intermediate to tobramycin and resistant to piperacillin, imipenem, and ciprofloxacin. The *P. aeruginosa*-NK2 isolate was sensitive to colistin, piperacillin, and tobramycin, intermediate to imipenem and resistant to cefepime, ciprofloxacin, and fosfomycin.

### Minimal inhibitory concentrations

The MICs and MBCs’ results were grouped, for the purpose of this study, according to their antimicrobial efficacies as high (≤ 30 mg/L), good (≥ 31 mg/L ≤ 60 mg/L), moderate (≥ 61 mg/L ≤ 90 mg/L), and no efficacy (≥ 91 mg/L). Five out of the eighteen metal ions tested showed high inhibitory antimicrobial activity (< 30 mg/L) against the two bacterial strains (Table 1). Platinum (7.8 mg/L), palladium, and tin (13.0 mg/L) ions demonstrated the best inhibitory antimicrobial activity against both *P. aeruginosa* isolates followed by the molybdenum ions (15.6 mg/L) and then gold ions (26.0 mg/L). The metal ions that showed good antimicrobial efficacy included rhenium and gallium (31.3 mg/L), rhodium (41.7- 52.1 mg/L), and aluminium (41.7 mg/L). The yttrium (62.5 mg/L), ruthenium (52.1 mg/L), tantalum (62.5 mg/L), indium (62.5 mg/L), zinc (62.5 mg/L), zirconium (83.3 mg/L), and niobium (83.3 mg/L) ions demonstrated moderate antimicrobial inhibitory effects, while no antimicrobial efficacy was demonstrated for the silver (104 mg/L) or copper ions (125 mg/L) against the bacterial isolates. Graphene (125 mg/L) and graphene oxide (> 500 mg/L) showed no antimicrobial activity. Among the 18 tested metal ions, only rhodium exhibited different MIC values against the two different isolates (41.7 mg/L *P. aeruginosa* NK1 and 52.1 mg/L *P. aeruginosa* NK2.

**Table 1:** MIC and MBC values (mg/L) (± SE) of metal ions, graphene, and graphene oxide against the bacterial strains

Metal/compound	Solvent	MIC		MBC	
		*P. aeruginosa* NK1	*P. aeruginosa* NK2	*P. aeruginosa* NK1	*P. aeruginosa* NK2
Y	HNO_3_ (2%)	62.5 ± 0.00	62.5 ± 0.00	125 ± 0.00	125 ± 0.00
Zr		83.3 ± 20.8	83.3 ± 20.8	333 ± 83.3	333 ± 83.3
Nb		83.3 ± 20.8	83.3 ± 20.8	333 ± 83.3	250 ± 83.3
Ag		104 ± 20.8	104 ± 20.8	125 ± 0.00	125 ± 0.00
Ta		62.5 ± 0.00	62.5 ± 0.00	83.3 ± 20.8	83.3 ± 20.8
I		62.5 ± 0.00	62.5 ± 0.00	125 ± 0.00	125 ± 0.00
Al		41.7 ± 10.4	41.7 ± 10.4	41.7 ± 10.4	41.7 ± 10.4
Cu		12.5 ± 0.00	125 ± 0.00	125 ± 0.00	250 ± 0.00
Zn		62.5 ± 0.00	62.5 ± 0.00	125 ± 0.00	125 ± 0.00
Re		31.3 ± 0.00	31.3 ± 0.00	31.3 ± 0.00	31.3 ± 0.00
Ga	HNO_3_ (5%)	31.3 ± 0.00	31.3 ± 0.00	83.3 ± 20.8	83.3 ± 20.8
Ru	HCl (5%)	52.1 ± 10.4	52.1 ± 10.4	62.5 ± 0.00	62.5 ± 0.00
Rh		41.7 ± 10.4	52.1 ± 10.4	62.5 ± 0.00	62.5 ± 0.00
Pt		7.8 ± 0.00	7.8 ± 0.00	7.81 ± 0.00	7.81 ± 0.00
Au		26.0 ± 5.20	26.0 ± 5.20	41.7 ± 10.4	41.7 ± 10.4
Pd		13.0 ± 2.60	13.0 ± 2.60	41.7 ± 10.4	41.7 ± 10.4
Mo	HCl (10%)	15.6 ± 0.00	15.6 ± 0.00	31.3 ± 0.00	31.3 ± 0.00
Sn		13.0 ± 2.60	13.0 ± 2.60	13.0 ± 2.60	13.0 ± 2.60
HNO_3_ (2%)		0.125%	0.125%	0.125%	0.125%
HNO_3_ (5%)		0.23%	0.23%	0.31%	0.31%
HCl (5%)		0.62%	0.62%	0.62%	0.62%
HCl (10%)		1.25%	1.25%	1.25%	1.25%
Graphene		125 ± 0.00	125 ± 0.00	250 ± 0.00	250 ± 0.00
Graphene oxide		> 500	> 500	> 500	> 500
Ethanol (50%)		> 500	> 500	> 500	> 500

### Minimal bactericidal concentrations

Platinum (7.8 mg/L) and tin (13.0 mg/L) ions demonstrated high bactericidal activity against the bacterial strains, whilst rhenium and molybdenum (31.1 mg/L), and gold and palladium (41.7 mg/L) ions showed good bactericidal activity (Table 1). Ruthenium, rhodium (62.5 mg/L), tantalum, and gallium (83.3 mg/L) ions demonstrated moderate activity. Yttrium, silver, zinc (125 mg/L), zirconium (333 mg/L) and copper ions (125 mg/L against NK-1 and 250 mg/L against NK-2) and niobium ions (333 mg/L against NK-1 and 250 mg/L against NK-2) demonstrated no bactericidal efficacy. The MBCs of graphene oxide (> 500 mg/L), and graphene (250 mg/L) also indicated no bactericidal activity. Despite the differences shown in their antibiotic profiles, both *P. aeruginosa* strains exhibited the same MBC values for graphene, graphene oxide, and each metal ion with the exception of niobium and copper.

### Fractional inhibitory concentrations

The FIC was carried out to determine the synergistic activity between the eight metal ions exhibiting the best antimicrobial activity with the graphene-based compounds in a 1:1 ratio. When combined with graphene oxide, silver, and yttrium ions showed an enhanced antimicrobial activity against both *P. aeruginosa* species (FIC ≤ 0.5). When tested with graphene, a synergistic effect was determined with molybdenum, silver, yttrium, and palladium ions against the two strains of *P. aeruginosa* (FIC ≤ 0.5). The platinum ion–graphene combination was the only one that demonstrated an antagonistic activity against *P. aeruginosa* isolates (FIC ≥ 4) (Table 2).

**Table 2:** FIC values of eight metal ions combined with graphene or graphene oxide against *P. aeruginosa* bacterial isolates

		Metal ions							
		Mo	Au	Ag	Sn	Y	Pt	Pd	Ru
Gr	*P. aeruginosa*—NK1	0.44	0.99	0.50	1.00	0.33	4.00	0.37	0.99
	*P. aeruginosa*—NK2	0.50	1.00	0.24	1.00	0.24	4.00	0.50	1.00
GO	*P. aeruginosa*—NK1	0.99	0.88	0.50	1.00	0.37	3.00	1.00	1.00
	*P. aeruginosa*—NK2	1.00	1.00	0.50	1.00	0.50	2.00	1.00	1.00

Metal ions and graphene oxide are considered to be synergistic if ∑FIC ≤ 0.5; additive if 0.5 < ∑FIC ≤ 1; indifferent if ∑FIC > 1 and antagonistic if ∑FIC ≥ 4

### Antimicrobial testing against biofilms

**Fig. 1:**
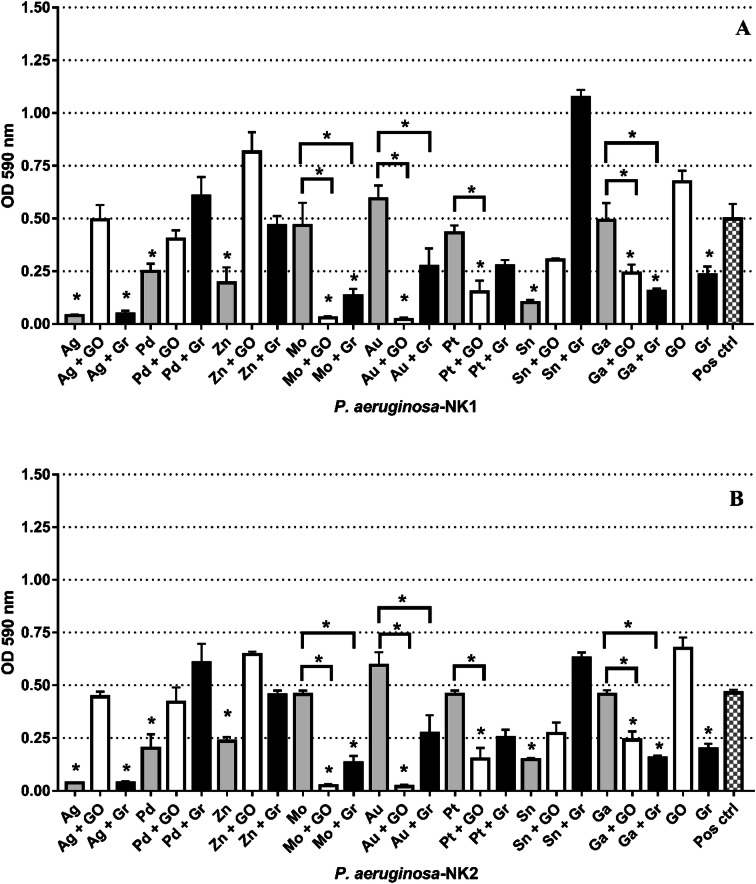
The effect of metal ions and graphene-based compounds on pre-formed biofilms of **a***P. aeruginosa*-NK1 and **b***P. aeruginosa*-NK2 after 24 h treatment. The absorbance values (± SD) are directly proportional to the amount of intact biofilm following treatment. Asterisk represents *p* < 0.05

The antimicrobial activity of the metal ions shown to be effective against the planktonic cells of *P. aeruginosa* strains were investigated against the biofilm forms of the bacterial strains. Out of the eight metal ions, tin, zinc, palladium, and silver ions were able to reduce the biofilm production of *P. aeruginosa* NK-1 and *P. aeruginosa* NK-2 (Fig. 1). Graphene showed an antimicrobial effect against the biofilm forms of the bacterial strains to a greater extent than the planktonic form (*p* < 0.0001). The optimal antimicrobial effect against the biofilms of the two isolates was demonstrated for the platinum–graphene oxide, gallium–graphene oxide, molybdenum–graphene oxide, gold–graphene oxide, silver–graphene, gallium––graphene and molybdenum–graphene combinations (*p* < 0.05) (Fig. 1).

**Fig. 2:**
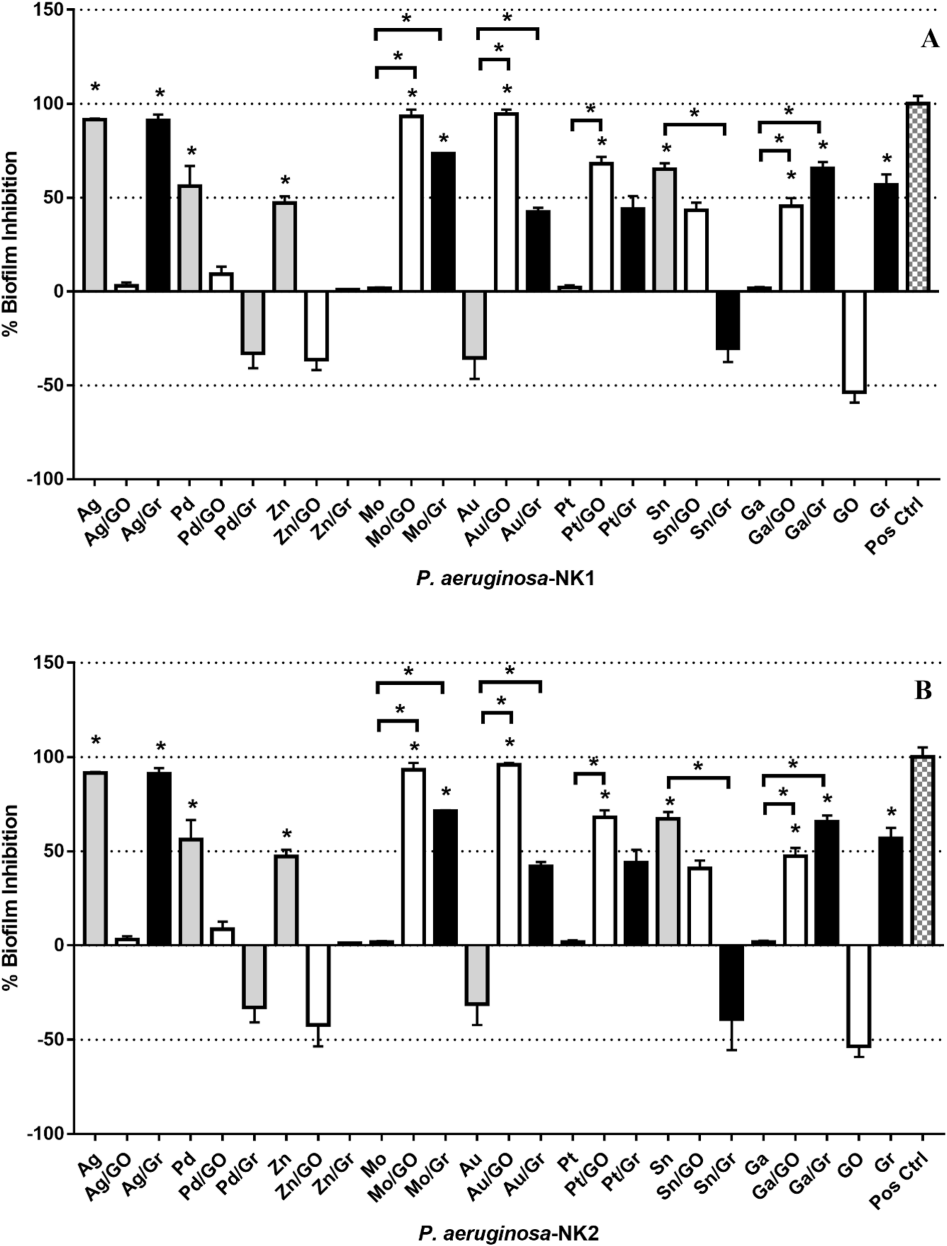
The antimicrobial effect of metal ions and graphene-based compounds on the metabolic activity of biofilm cells of *P. aeruginosa*: **a***P. aeruginosa* NK1 and **b***P. aeruginosa* NK2 isolates as assessed by percentage reduction in XTT colorimetry. Data shown as mean (± SD). The dotted red line signifies the threshold for minimum antibacterial activity

The XTT assay showed that the same metal ions and compounds that inhibited the biofilm were able to reduce their metabolic activity. The following combinations Au–GO (94%), Mo–GO (93%), Ag (91%), Ag–Gr (91%) showed significantly reduced metabolic activity of the viable biofilm cells of both isolates. The combined results from the biofilm and XTT assays showed that the addition of graphene or graphene to selected metal ions enhanced their antimicrobial effect compared to the compounds or metal ions alone. No variance in antimicrobial activity tested via CVBA and XTT was noted between the two tested isolates, although they both exhibited different antibiotic profiles (Fig. 2).

## Discussion

Following the emergence of antibiotic-resistant bacteria causing healthcare associated and community-acquired infections, there is a need for effective antibacterial and biofilm treatments (Gold et al. 2018). Currently, *Pseudomonas* spp. is the third most prominent cause of Gram-negative bloodstream infections responsible for 4% of hospital-acquired bacteraemia cases (Gold et al. 2018). As such, adequate control of *P. aeruginosa* infections represents a substantial challenge for clinicians.

### Antibiotic susceptibility testing

Each *P. aeruginosa* isolate used in this study was resistant to at least two classes out of the eight classes of antibiotics generally used to treat *P. aeruginosa* infections (Bassetti et al. 2018). While the treatment of *P. aeruginosa* depends on the clinical isolate itself, burn units alternate between various topical antimicrobials for *P. aeruginosa* to reduce the risk of resistance (Japoni et al. 2009).

### Antimicrobial efficacy of single metal ions

The results demonstrated that molybdenum, tin, platinum, palladium, and gold ions showed the greatest antimicrobial activity against *P. aeruginosa* NK-1 and NK-2 in the planktonic form to different degrees. The degree of antimicrobial efficacy towards a specific pathogen may be affected by the surrounding milieu or the bacteria itself, or the specific chemical properties of the metal ions, for example, their reduction potential and speciation. Thus, it may be prudent to use different metal ions tailored to a specific bacteria and environment.

Overall, the same values for the MIC and MBC for the metals, graphene, and graphene oxide were recorded for the two distinct isolates of *P. aeruginosa* despite having different antibiotic profiles. The only exceptions were niobium and rhodium that displayed different MICs and MBCs against the two strains. This demonstrated that the metal ions were able to inhibit or kill the bacterial planktonic or biofilm cells of *P. aeruginosa* isolates regardless of their antimicrobial resistance profiles.

It has been reported that metal ions are capable of disrupting cell growth cycles in a concentration dependent manner, and that this is due to the physical and chemical properties of both the metals and their accessibility to donor ligands within the intracellular bacterial biomolecules (Lemire et al. 2013). This might partly explain the observation that molybdenum, tin, platinum, and palladium ions, being classified as “soft metals”, showed the highest antimicrobial efficacy against the bacterial isolates. These metals have high electronegativity values and are able to form covalent bonds, with preferential binding of nitrogen or sulphur in the proteins of *P. aeruginosa*. It has been reported that the antibacterial toxicity of these metals is proportional to their affinity for sulphur (Lemire et al. 2013).

### Antimicrobial efficacy of graphene and graphene oxide

Graphene-based compounds have emerged recently as potential novel broad-spectrum antibacterial agents (Hu et al. 2010; Tu et al. 2013; Slate et al. 2018). Several studies have indicated that graphene oxide and reduced graphene oxide inhibited the growth of *Escherichia coli* and *P. aeruginosa* with at least an 86–99.9% reduction in viability (Hu et al. 2010; Tu et al. 2013). However, this was inconsistent with our results where graphene showed only a moderate effect (MIC = 125 mg/L) and graphene oxide (MIC > 500 mg/L) showed no inhibitory effect against any of the isolates tested. On the other hand, Ruiz et al. have previously demonstrated that graphene oxide enhanced bacterial growth (Ruiz et al. 2011). However, the bactericidal effect of graphene-based composites remains poorly understood. This requires further investigation, especially since their effect on the structure and viability of bacterial cells has also been shown to be dependent on the sample preparation and concentration, time of exposure, and physico-chemical properties, as well as on the method of microbiological analysis (Akhavan and Ghaderi, 2010; Hu et al. 2010). It may be speculated that in this study, the negative antimicrobial results obtained with graphene oxide against the planktonic bacterial form may be due to the type of graphene oxide used. It has been demonstrated that highly purified graphene oxide was inert against planktonic forms of bacterial cells (Barbolina et al. 2016). Furthermore, Barbolina et al. (2016), assigned the bacteriostatic and bactericidal effects of graphene oxide found in previous studies to the contaminants (sulphur, nitrogen) that remained following the preparation of the graphene oxide rather than to the effects of the graphene oxide itself (Barbolina et al. 2016).

### Synergy testing between graphene-based compounds and metal ions

Despite the fact that neither graphene oxide or silver ions alone showed any antimicrobial activity against any of the isolates, the combination of graphene oxide or graphene with the molybdenum, tin, platinum, silver, or palladium ions together enhanced their bacteriostatic activity, and thus, the addition of graphene or graphene oxide resulted in a synergistic effect. This may indicate that the metals/graphene combinations resulted in complementary modes of action, which enhanced the total antimicrobial efficacy, although this needs further investigation. A possible hypothesis might be that the metal ions, being in solution, become evenly dispersed in the environment surrounding the pathogen with no specific laterality (McQuillan et al. 2012). Meanwhile, adsorption of graphene particles by the pathogen may induce cell wall depolarization making the bacterial cell more permeable to destruction, assisting the metal ions to enter inside the bacterial cell (Pal et al. 2007).

The synergistic antibacterial effect of graphene or graphene oxide with the metal ions was independent of the antibiotic resistance pattern of any of the isolates. This might be due to the difference between the targeted mode of action of the antibiotics and the more general mechanism of metal ions (Kohanski et al. 2010).

### Antimicrobial efficacy of the tested compounds against biofilms

While it has been estimated that 65% of bacterial infections are associated with biofilms (comprising both device and non-device associated infections) (Jamal et al. 2018), four metal ions and seven combinations tested reduced the amount of a 7 day biofilm of both the AMR *P. aeruginosa* isolates. While silver alone and graphene alone exhibited no effect against the planktonic forms of the isolates, when the silver–graphene combination was tested, it reduced both the number of planktonic cells and the amount of biofilm formed. Graphene oxide showed reduced growth of the biofilms when combined to molybdenum, gold, platinum, or gallium. The assessment of the cell viability of the biofilms of both isolates post-treatment with silver, silver–graphene, gold–graphene oxide, and molybdenum–graphene oxide showed a significant reduction in biomass.

## Conclusions

This study demonstrated that molybdenum, platinum, gold, tin, and palladium ions exhibited antibacterial properties against *P. aeruginosa* strains regardless of their resistance profiles. The antimicrobial effect of the key metal ions was more enhanced when combined with either graphene or graphene oxide. Furthermore, the resulting combinations (silver–graphene, molybdenum–graphene/graphene oxide, gold/graphene/graphene oxide, platinum/graphene/graphene oxide, and gallium/graphene/graphene oxide) were able to significantly reduce the biofilm formation of the AMR isolates. Overall, the silver–graphene and molybdenum–graphene showed an antimicrobial effect against both planktonic and biofilm forms of the bacteria irrespective of their antibiotic resistance profiles. In light to the increasing resistance to the currently used antibiotics, this study suggests that metal ions and graphene derivatives present a potential biocidal alternative for the control of AMR *P. aeruginosa* infections for potential use in burn wounds.

## Data Availability

The data sets generated during and/or analysed during the current study are available from the corresponding author on reasonable request.
